# Osteophyte-Sparing Treatment of Mucous Cysts: Case Analysis and Surgical Technique

**DOI:** 10.5435/JAAOSGlobal-D-21-00164

**Published:** 2021-11-04

**Authors:** Taylor Bates, Julia A.V. Nuelle, James K. Aden, Gary G. Wind, Thomas B. Lynch, Thomas R. Shepler

**Affiliations:** From the Department of Orthopaedic Surgery, San Antonio Military Medical Center, Fort Sam Houston, TX (Dr. Bates, Dr. Nuelle, and Dr. Lynch); San Antonio Military Medical Center, San Antonio Uniformed Services Health Education Consortium, Fort Sam Houston, TX (Dr. Aden); Department of Surgery, Uniformed Services University of the Health Sciences, Bethesda, MD (Dr. Wind); and Manus Center PA, Vienna, VA (Dr. Shepler).

## Abstract

The purpose of this study was to examine patient outcomes and the risk of recurrence of mucous cysts affecting the distal interphalangeal (DIP) joint in the absence of osteophytectomy using the described flap technique.

**Methods:**

A retrospective review of 143 records of patients who were treated for mucous cysts of the DIP joint by a single surgeon. Inclusion criteria included the absence of an osteophytectomy during treatment using the described dorsally based flap technique and a minimum of 12 months of follow-up.

**Results:**

A total of 143 mucous cysts affecting the DIP joint of 131 patients with an average age of 65.3 years were included. The average follow-up was 21.9 months (12 to 139). Postoperative DIP joint extension was less in the surgical digit compared with the same digit of the contralateral hand with a significant change from the preoperative motion (1.5° versus 0.3°; *P* = 0.05). No significant change in the postoperative flexion of the DIP joint was observed compared with that of the contralateral side (−1.4° versus −0.9°; *P* = 0.57). Recurrence occurred in 2 patients (1.4%). No infections or wound complications were identified.

**Conclusions:**

Using the described technique without an osteophytectomy seemed to be an effective treatment of mucous cysts originating from the DIP joint.

Cysts affecting the distal interphalangeal (DIP) joint were described as early as 1883 by Hyde as synovial lesions of the skin and are known today by a variety of names including myxomatous degeneration cysts, recurring myxomatous cutaneous cysts, and mucous cysts.^1-3^ Mucous cysts of the DIP joint are similar to other ganglion cysts in that they have a connecting stalk that communicates with the nearby joint. These are common lesions associated with degenerative changes and typically occur in middle age and late adulthood. They commonly arise through the interval between the collateral ligament and extensor mechanism but will occasionally arise simultaneously on both sides of the tendon (ie, extensor mechanism) or rarely through the ligament or tendon (Figures 1 and 2).

**Figure 1:**
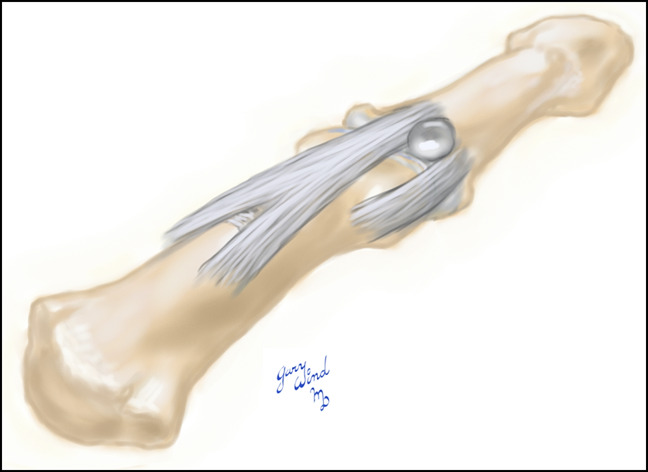
Illustration demonstrating a cyst originating from the area between the extensor mechanism and collateral ligament.

**Figure 2:**
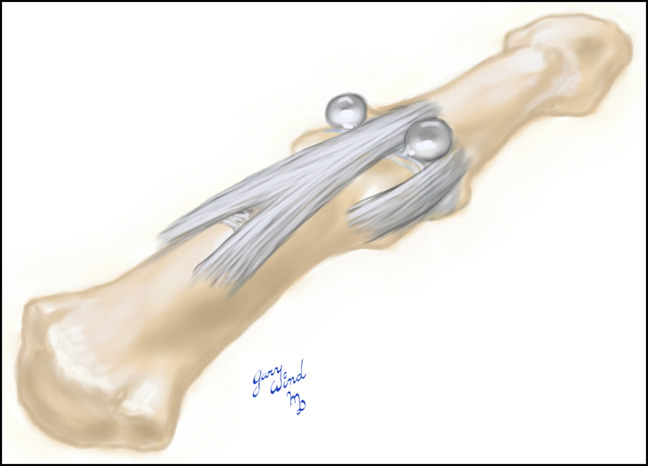
Illustration demonstrating a dumbbell cyst arising from both sides of the extensor mechanism and between each collateral ligament.

Despite the almost universal acceptance of the need for an osteophytectomy when treating these lesions,^5-7^ some authors have noted low recurrence rates when treating ganglion cysts with techniques that do not include bony débridement. Furthermore, osteophyte débridement risks injury to the germinal matrix and has the potential to result in a nail deformity.^4^ The purpose of this article was to examine the patient outcomes and risk of recurrence using the described technique that excludes bony débridement and is based on the experience of the senior author (T.R.S.).

## Methods

After Investigational Review Board approval, 514 records of patients who had undergone mucous cystectomy by a single surgeon were examined. Patients were excluded if they had undergone an osteophytectomy, if their record was incomplete, or if they were not followed up for a minimum of 12 months. Exclusion of an osteophytectomy became a routine practice for the senior author as he adopted the described technique. The minimum follow-up time was supported by Fritz et al^8^ who noted that all recurrences in their series occurred within 12 months. All surgical excisions and postoperative examinations were done by the original surgeon (T.R.S.). Surgical records were searched for patients who had undergone mucous cystectomies using the described technique while sparing the osteophyte, and the following data were collected: age, sex, laterality, digit involved, presence of nail deformity, recurrence, complications (ie, wound necrosis), need for repeat procedures, and range of motion. Of note, if hyperextension of the joint was present, DIP joint extension was recorded as zero degree in the original record.

Categorical data were summarized using percentages. Means and standard deviations were used for summary statistics of continuous variables. Changes in preoperative and postoperative active ROM flexion and extension of the DIP were also analyzed using paired *t*-test. Significance for results was established when *P* values were less than 0.05. All statistical analysis was performed using JMP v13.2 SAS Corp.

### Surgical Technique

**Figure 3:**
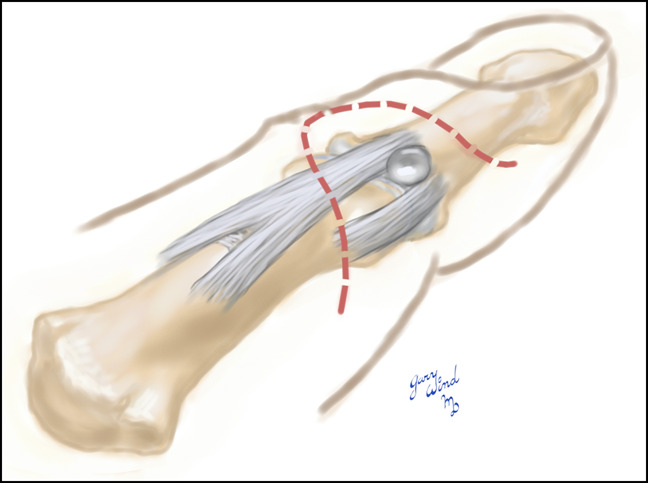
Illustration demonstrating the planned surgical incision dorsal to the mid-axis with the cyst located at the base of the flap.

Anesthesia is achieved with a digital block and hemostasis maintained with a tourniquet. A flap is planned with the lesion at the flap's base (Figure 3). Proximally, the incision is planned dorsal to the mid-axis line to preserve as much blood supply to the flap as possible and prevent tip necrosis. The absolute extent of the flap is from mid-axis to mid-axis, although this is rarely needed. The flap is made long enough to extend to the opposite side of the extensor mechanism so that the interval between the extensor mechanism and the collateral ligament can be explored on each side. This allows access to the joint on both the radial and ulnar sides of the extensor tendon to reliably identify the location of cyst emanation. The same incision can also be used to treat a mucous cyst that recurs on the opposite side of the extensor tendon. The plane of the flap is found proximal to the distal extension crease by blunt dissection with scissors and remains superficial to the lateral bands as dissection is done distally. Care is taken not to dissect beyond the volar mid-axis because this is not necessary and can put the skin flap at risk for necrosis. Although it can be released without consequence, identification and preservation of Cleland's ligament at the base of the flap can help the surgeon avoid excessive volar dissection toward the neurovascular bundle. The distal portion of the flap lying between the distal extensor crease and the distal eponychial edge of the skin has no dissection plane, and the surgical plane must be created with a scalpel taking care to not enter the germinal matrix of the nail plate.

**Figure 4:**
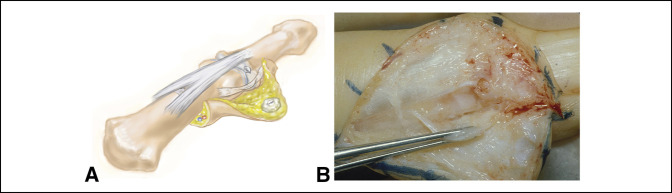
**A** and **B**, Illustration and intraoperative photographs, respectively, demonstrating exposure obtained by exposing the interval between the extensor mechanism and the collateral ligament.

**Figure 5:**
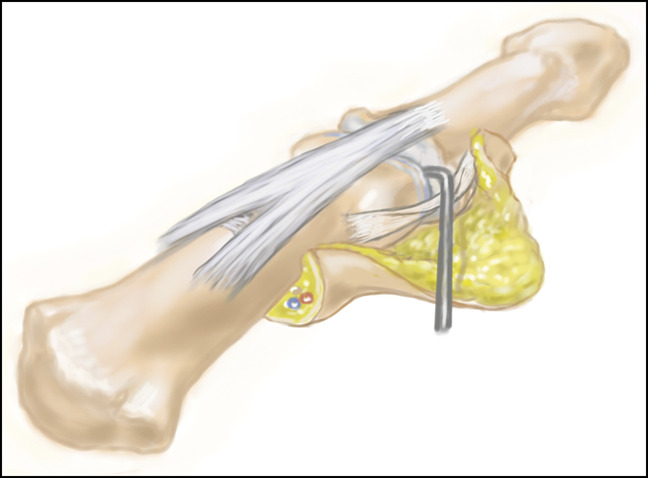
Illustration demonstrating a dental probe being used to ensure joint exposure with partial or complete takedown and reflection of the proximal portion of the collateral ligament and adjacent joint capsule.

The capsulotomy is started by making a longitudinal incision along the edge of the extensor mechanism at the level of the DIP joint. This is performed by orienting the blade parallel and adjacent to the metaphysis of the middle phalanx, skiving the knife along the side of the distal condyle, thereby partially or completely detaching the proximal portion of the collateral ligament and leaving it in situ. The collateral ligament is then partially released distally from the base of the distal phalanx, taking care not to enter the germinal matrix. The collateral ligament along with its adjacent joint capsule is kept in the base of the flap. No portion of the collateral ligament is excised but left in situ in the base of the flap to allow for reattachment through scar formation. The released collateral ligament is retracted with a skin hook, and a dental probe is passed around the condyle of the middle phalanx to ensure release of this proximal attachment and capsule (Figure 4, A and B and 5). Care must be taken to avoid complete release of the proximal portion of the collateral ligament when operating on the ulnar side of the thumb or the radial side of the index finger to ensure no instability during pinch is created. In these cases, the volar one-third of the collateral ligament is left intact. The cyst itself is not excised from the flap to avoid risking the flap's integrity. The wound is then closed in a standard fashion and a dressing applied. Resumption of motion is started 1 week postoperatively. Sutures are removed 14 days postoperatively.

## Results

A total of 143 mucous cysts affecting the DIP of 131 patients (82 women and 49 men) with an average age of 65.3 years (range 41 to 90) were included in the analysis (Table 1). The average follow-up was 21.9 months (range 12.0 to 139.0). The left hand was involved in 57 patients (39.9%) and the right hand in 86 patients (60.1%). Recurrence occurred in two patients (1.4%). One patient had recurrence at 16 months without further intervention documented. The second patient had recurrence at 38 months and underwent revision excision with a documented follow-up of 88 months without cyst return. Preoperative nail deformities such as grooving or linear flattening were present in 58 of the 143 (40.6%) patients, which resolved in 55 patients (94.8%). No complications or reports of instability were noted.

**Table 1:** Patient Demographics

Sex	Laterality	Digit	Recurrence
Female = 90 (62.9%)	Left = 57 (39.9%)	1st = 29 (20.3%)	
		2nd = 38 (26.6%)	
Male = 53 (37.1%)	Right = 86 (60.1%)	3rd = 60 (42.0%)	2 (1.4%)
		4th = 7 (4.9%)	
		5th = 9 (6.3%)	

Preoperative and postoperative active ROM of the DIP was compared with the same digit of the contralateral hand. In addition, the change in preoperative and postoperative motion were compared (Table 2). The average preoperative ROM of the surgical digit of the DIP joint was from 1.3° (zero degree to 23°) to 67.6° (37° to 92°) of flexion. The average preoperative ROM of the contralateral comparison digit for the DIP joint was from 1.2° (zero degree to 33°) to 69.9° (38° to 90°) of flexion. No statistically significant difference was found in the preoperative extension of the surgical digit compared with that of the contralateral digit (1.3° versus 1.2°; *P* = 0.854). Preoperative flexion was significantly less in the surgical digits compared with that in the contralateral digits (67.6° vs 69.9°; *P* ≤ 0.050).

**Table 2:** Range of Motion Comparison Between the Surgical Digit and Contralateral Side

	Surgical Digit	Contralateral Side	*P*
Preoperative DIP joint extension	1.3° ± 4.2°	1.2° ± 4.5°	0.854
Preoperative DIP flexion	67.6° ± 9.2°	69.9° ± 9.3°	<0.050
Postoperative DIP extension	2.8° ± 5.9°	1.5° ± 4.3°	<0.050
Postoperative DIP flexion	66.3° ± 9.8°	69.0° ± 9.2°	<0.050
Change in DIP extension	1.5° ± 4.9°	0.3° ± 3.1°	<0.050
Change in DIP flexion	−1.4° ± 7.4°	−0.9° ± 7.3°	0.572

DIP = distal interphalangeal

The average postoperative ROM of the surgical digit for the DIP joint was from 2.8° (zero degree to 27°) to 66.3° (37° to 86°) of flexion. The average postoperative ROM of the contralateral comparison digit was from 1.5° (zero degree to 20.0°) to 69.0° (38° to 86°) of flexion. Postoperative extension was significantly less in the surgical digit (2.8° versus 1.5°; *P* ≤ 0.050). Postoperative flexion remained significantly less in the surgical digits (66.3° versus 69.0°; *P* ≤ 0.050). The change in preoperative and postoperative extension was significantly more in the surgical digit (1.5° versus 0.3°; *P* ≤ 0.050). On average, both surgical and contralateral digits lost flexion. However, no significant change in flexion of the surgical side was observed compared with the contralateral digit (−1.4° versus −0.9°; *P* = 0.572).

## Discussion

Mucous cysts of the DIP are challenging to treat. Their high rate of recurrence has led to the development of various therapies and surgical techniques. Previously described treatments include repeated needle punctures, cauterization, steroid injection, proteolytic injection, radical amputation, irradiation, skin grafting, flap coverage, dissection with an osteophytectomy, and dorsal capsulectomy.^4,5,7,9,10^ Needle aspiration with or without injection with local anesthetic and steroid have recurrence rates ranging from 40% to 100%, whereas simple excision recurrence has been reported to range from 25% to 28%.^6,9,11-13^ Clinical studies have shown much lower recurrence rates when osteophytes are removed. Eaton et al^5^ reported 1 recurrence in 50 patients (2%), Kleinert et al^7^ reported no recurrence in 36 patients, and Rizzo et al^6^ reported no recurrences in 54 patients with their respective techniques, all three emphasizing an osteophytectomy.

The importance of performing an osteophytectomy was emphasized in techniques with high rates of success.^5,7,14,15^ However, Kanaya et al^4^ noted that excision of the osteophyte requires disruption of the dorsal joint capsule. Despite the similarity in pathogenesis with wrist ganglion cysts, capsular débridement, not bony débridement, is an effective treatment of ganglion cysts of the wrist.^16^ The results of this study suggest that this is also true for mucous cysts of the DIP joint and that débridement of the stalk's emanation by the wide opening of the joint capsule results in scar formation that seals the pathologic area of the capsule. The findings of this study support the conclusion by Kanaya et al^4^, that bony débridement is not a critical step to achieve a low rate of recurrence.

The recurrence rate of 1.4% (2/143) is comparable with the 2% (1/50) rate reported by Eaton et al^5^ who reported a minimum follow-up time of 6 months and concluded osteophytectomy is required to prevent recurrence. Although the cause for recurrence in this study is difficult to elucidate, one of the two patients had documentation of the cyst arising simultaneously on both sides of the extensor mechanism. Therefore, this recurrence may be explained by inadequate capsulotomy. Despite these two recurrences, the final recurrence rate of less than 1.5% may be acceptable and comparable with other techniques.

Time of recurrence after surgical excision varies significantly between studies. Fritz et al^8^, who recorded a cyst recurrence rate of 3.4% (3/86) after surgical excision, noted all three of their cyst recurrences occurred within 12 months of excision. However, Kasdan et al^17^ noted two recurrences (1.8%) that presented at 41 and 70 months after surgery in their study of 113 patients with a minimum follow-up of 6 months. Therefore, recurrence may occur well beyond 12 months and could be underestimated with available literature. However, a minimum follow-up time of 12 months is comparable with other studies documenting outcomes after treatment of these lesions.^4,5,8,9,17^

Although the described technique can include an osteophytectomy if the surgeon chooses, avoiding osteophyte excision can be advantageous. Performing an osteophytectomy risks inadvertent débridement of the germinal matrix, which can lead to a new or persistent nail deformity. In cases of infection, which occurred in 2.3% of patients in one study^8^, the same exposure can be used for joint débridement or to prepare the joint for arthrodesis, and the flap can be left open to allow the joint to drain.

Joint stiffness and diminished DIP joint ROM were reported after cyst excision, and these changes worsened after osteophyte débridement.^4,8,15^ In this study, preoperative flexion was less in the surgical digit compared with the contralateral side and remained significantly less at the last follow-up. A significant loss of extension was noted compared with the contralateral side. However, the differences in flexion and extension preoperatively and postoperatively are small (average ≤1.5°). Ultimately, these findings are relevant because they suggest that the described technique is unlikely to lead to a clinically significant loss of motion.

Limitations to this study include the lack of randomization; no reported postoperative pain scores; and all procedures being done by the same surgeon, which may limit external validity and create selection bias. The strengths of this study include a large number of patients; postoperative examinations and ROM measurements done by the original surgeon; and the long follow-up times with range of motion data (average 21.9 months). This study supports the use of the described flap technique with osteophyte sparing for the treatment of mucous cysts affecting the DIP joint.
